# High exposure to phthalates is associated with HbA1_c_ worsening in type 2 diabetes subjects with and without edentulism: a prospective pilot study

**DOI:** 10.1186/s13098-022-00875-0

**Published:** 2022-07-20

**Authors:** Alessandro Mengozzi, Fabrizia Carli, Samantha Pezzica, Edoardo Biancalana, Amalia Gastaldelli, Anna Solini

**Affiliations:** 1 Department of Clinical and Experimental Medicine, University of Pisa, Pisa, Italy; 2 Sant’Anna School of Advanced Studies, Pisa, Italy; 3 Institute of Clinical Physiology, National Research Council, Pisa, Italy; 4 Department of Surgical, Medical, Molecular and Critical Area Pathology, University of Pisa, Pisa, Italy

**Keywords:** Phthalate, Cardiovascular risk, Diabetes, Biomarker, Edentulism

## Abstract

**Background:**

Phthalates exposure and complete edentulism are related to both low socioeconomic status. No study by far has verified if and to what extent these two conditions are related. We aimed to explore their potential association and interplay in the metabolic control and cardiovascular risk profile.

**Methods:**

In our small (n = 48) prospective pilot study twenty-four patients with type 2 diabetes (DnE) and twenty-four patients with type 2 diabetes and edentulism (DE) followed for 19 ± 2 months were treated according to best clinical standards. Phthalates’ exposure was evaluated by urinary concentration of di-2-ethylhexylphthalate (DEHP), metabolites, *i.e.* mono 2-ethylhexyl phthalate (MEHP), mono-2-ethyl-5-oxohexyl phthalate (MEOHP) and mono 2-ethyl-5-hydroxyhexyl phthalate (MEHHP).

**Results:**

No association between phthalates and edentulism was found, nor did edentulism affect glucose control. Higher phthalates exposure was associated with a glycated haemoglobin worsening. This association was found for all the measured phthalates metabolites, both as a whole (DEHP; r = 0.33, p = 0.0209) and individually: MEHP (r = 0.41, p = 0.0033), MEHHP (r = 0.32, p = 0.028), MEOHP (r = 0.28, p = 0.0386).

**Conclusions:**

Phthalates are not associated with edentulism but predict the worsening of glucose control in subjects with type 2 diabetes. These findings might prove relevant in identifying novel biomarkers of cardiometabolic risk. Further studies are needed to validate our results and estimate the true potential of phthalates in terms of risk assessment.

## Background

Phthalates are environmental and dietary contaminants with a wide array of use. In the plastic industry, their primary use is to give flexibility to polyvinyl chloride (PVC) polymers [1]. Given that they are quickly released from materials, people are intensively exposed to these compounds through diet, inhalation, and dermal absorption [2, 3]. The daily contact with phthalate-containing items is, thus, widespread among the general population. As recent national and international health biomonitoring studies show [4–6], exposure to phthalates is highly variable also within the national borders [7]. It is associated with a worse sociodemographic condition, like the rural environment or the lower level of education [8]. The issue of such associations lies in their implicit consequences on cardiometabolic health. Indeed, due to their ability to interfere with hormone signaling, phthalates act as endocrine disruptors. Several large epidemiological observations have shown their detrimental effect on cardiometabolic health. Obesity, insulin resistance, type 2 diabetes (T2D), albuminuria and atherosclerosis, all conditions at high cardiovascular (CV) risk, have all been found to be associated with phthalates [9, 10].

Cardiovascular risk prevention policies should better consider the substantial contribution of socioeconomic disparities to CV disease prevalence. Health resources should be adequately distributed to fill the higher-income population gap by targeting modifiable CV risk factors like environmental exposure to phthalates. Indeed, their potential and harmful interaction with other determinants of cardiometabolic disease already present in the low demographic status’ population might further increase the CV disease-related burden that healthcare systems face in the twenty-first century [11]. Edentulism (*i.e*. the partial or complete loss of all the natural teeth) is, for instance, another major determinant of cardiovascular risk [12] and is involved in several impaired metabolism conditions, like T2D [13, 14] and chronic kidney disease [15]. Edentulism and tooth loss are also related to lower education [16], rural areas [17] and low socioeconomic position [18]. While some studies have reported the association of phthalates’ levels with bad oral health [19], no studies have, so far, explored the potential association between phthalates and edentulism, nor their relative contribution to CV risk and other cardiometabolic health’s determinants, as body mass index (BMI) and glucose control. Several observations have described the presence of phthalates in some orally administered drugs [20, 21], further exposing the oral cavity to their potentially harmful effect. Phthalates have been found in personal oral care products [22] and in drug delivery systems for periodontitis treatment [23]. Thus, it is relevant to investigate if these compounds might promote inappropriate oral health control.

We aimed to explore the potential association between DEHP exposure and edentulism in humans and their interplay in metabolic control and cardiovascular risk. To this scope, we designed a prospective pilot and hypothesis-generating study to investigate if exposure to phthalates and edentulism in a small cohort of T2D patients were related to worse glycemic control and whether this could affect cardiovascular health risk.

## Methods

### Subjects

Among the patients consecutively referring to our Diabetes Outpatient’s clinic (S. Chiara Hospital, University of Pisa, Pisa, Italy), we recruited twenty-five T2D subjects (DnE group) and twenty-five subjects with T2D and complete edentulism (DE group). Complete edentulism was defined as the physical state of the jaw(s) following removal of all erupted teeth and the condition of the supporting structures available for reconstructive or replacement therapies [24]. Inclusion criteria were: T2D diagnosis (according to American Diabetes Association criteria [25]), diabetes duration ≥ 12 months, eGFR > 30 mL/min/1.73 m^2^; age 50–80 years. Exclusion criteria were: individuals on diuretics (to avoid phthalates’ exposure confounders, as previously described [26], severe obesity (BMI > 40 kg/m^2^), occurring acute complications/comorbidities and presence of severe cardiovascular, pulmonary, hematologic or hepatic disease. People taking medications other than anti-hypertensive, anti-diabetic and lipid-lowering drugs were also excluded from the study. The study protocol was approved by the Tuscany Ethics Committee for Clinical Research, and participants provided written informed consent.

### Study protocol

Enrolled participants who agreed to participate underwent a baseline clinical visit between October 2019 and January 2020 at our Diabetes clinic, as per routine good standard care, followed by an outpatient visit each 6-month. In particular, the glucose control target was HbA1c of 48–52 mmol/mol (based on the whole clinical phenotype). The total duration of the study was October 2019-September 2021. During each clinical visit, the characteristics of the study population were obtained: age, sex, height, weight, BMI, smoking status (classified non- or past smoker vs current smoker), T2D durations, current therapies and blood pressure. The presence of CV disease (non-fatal acute myocardial infarction, non-fatal stroke, unstable angina and revascularization) was adjudicated by clinical records. Each participant was investigated for a familiar history of CV disease, T2D, periodontal disease, and dyslipidaemia during each visit. The day after the visit, routine blood laboratory tests were centrally performed after 12-h of fasting. eGFR was calculated using the CKD-EPI formula, LDL cholesterol was estimated by the Friedewald formula. The day after the baseline visit, patients also provided a single spot fasting urine sample collected between 08.00 and 10.00 a.m. in phthalate-free containers to determine the phthalates urinary metabolites. After collection, they were transported to the laboratory on ice and then stored at − 80 °C before being analyzed. Patients were treated according to good clinical practice recommended by the international guidelines. Moreover, they were instructed by the physician performing the visit to avoid further exposure to phthalates: written memoranda were provided to instruct the participant on common sources of phthalates’ exposure (*e.g.* packaged food, plastic cutlery, microwave oven cooked meals, long conservation products, pesticides-treated products).

### Phthalates’ exposure assessment

In the spot urine samples, the three major urinary metabolites of di-2-ethylhexylphthalate (DEHP), *i.e.* mono 2-ethylhexyl phthalate (MEHP), mono-2-ethyl-5-oxohexyl phthalate (MEOHP) and mono 2-ethyl-5-hydroxyhexyl phthalate (MEHHP), were analyzed by a method developed for U-HPLC/LCMS Quadrupole Time-of-Flight (QTOF) during the activities of the LIFE-PERSUADED project for the “human biomonitoring of phthalate and Bisphenol A” (3, 20), and validated during proficiency test (ICI/EQUAS) of the European Human Biomonitoring Initiative-HBM4EU project (https://www.hbm4eu.eu). We decided to selectively observe DEHP metabolites based on previous works reporting their relationship with metabolic diseases [7, 10]. Urinary samples (500 µl) were first deconjugated (using Abalonase purified β-Glucuronidase, UCT, Bristol, PA) for one hour at 60 °C, then purified by solid-phase extraction (SPE cartridge C18 ODS 3 Agilent Santa Clara, CA, USA) and injected for detection and quantification in the U-HPLC/LCMS system coupled with electrospray ionization (Agilent 1290 infinity and Agilent 6545 Santa Clara CA, USA) equipped with Agilent ZORBAX SB-Phenyl column (2·1 × 100 mm, 1·8-Micron). DEHP metabolites were quantified using labelled internal standards MEHP ^13^C_4_, MEOHP ^13^C_4_ MEHHP ^13^C_4_ (purchased from Cambridge Isotope Laboratories, Tewksbury, MA, USA) added to the samples before purification by C18 SPE. The sum of DEHP urinary toxic metabolites (ΣDEHP) was then calculated as the sum of its urinary metabolites. Limit of Detection and Quantification (LOD and LOQ) were determined according to analytical detection limit guidance by IUPAC and were: for MEHP 0.28 and 0.58 ng/ml, for MEOHP 0.18 and 0.48 ng/ml, for MEHHP 0.11 and 0.24 ng/ml, respectively, as previously reported [10].

The formation of the three DEHP metabolites analyzed in this study occurs in a stepwise metabolic pathway [27]. DEHP is first converted to MEHP (1^st^ step), and then MEHP hydroxylation to MEHHP (2nd step) followed by MEHHP oxidation to MEOHP (3rd step). Thus, we also investigated the relative metabolic rate (RMR) of the formation of DEHP metabolites. The first relative metabolic rate (RMR_1_) represents the rate of MEHP hydroxylation to MEHHP. The second relative metabolic rate (RMR_2_) represents the rate of MEHHP oxidation to MEOHP. RMRs were calculated as previously described [28]. Briefly, each DEHP metabolite concentration was converted in molar concentration, and the conversion rate was estimated according to the following formulas: RMR_1_ = [MEHHP]/[MEHP]; RMR_2_ = ([MEOHP]/[MEHHP]).

### Statistical analysis

Statistical analysis was performed using JMP^®^Pro 11.2 software (SAS Institute Inc., Cary, NC). Data are reported as mean ± standard deviation or median [1st quartile; 3rd quartile]. All continuous variables were tested for normality via Kolmogorov-Smirnoff-Tests and normalized via logarithmic transformation before appropriate analysis. Statistical comparisons for continuous variables were made using Student`s t-tests for continuous variables normally distributed, Mann–Whitney–Wilcoxon tests for continuous variables non-normally distributed and Chi-square tests for categorical variables. Differences between means were tested through one-way ANOVA and repeated measures ANOVA when groups were independent or dependent, respectively. Correlations between variables were tested using Pearson or Spearman rank correlations as appropriate. A sample size of 50 subjects was calculated to provide at least 83% power and an α level of 0.05 to detect a significant difference in the urinary level of DEHP metabolites. Phthalates potentially harmful levels were defined based on previous studies [10, 19, 26].

## Results

### Baseline population characterization

**Fig. 1:**
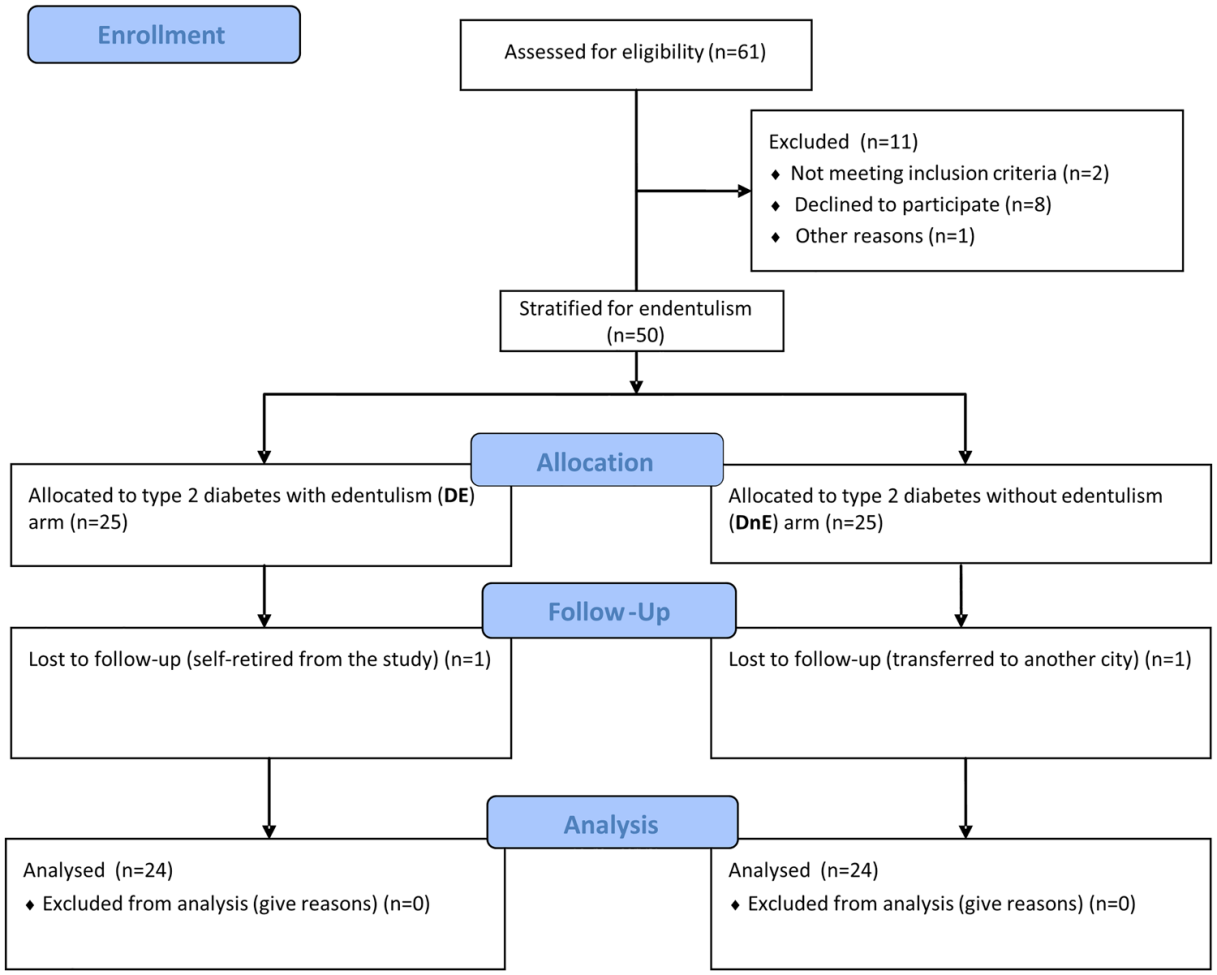
CONSORT flow diagram of the study

Two patients (one for each group) were lost at follow-up for personal reasons. A total of forty-eight subjects completed the protocol (Fig. 1). No death occurred during the follow-up period. No participant was put on steroidal or non-steroidal anti-inflammatory drugs for more than three days/month. The study population’s characteristics are shown in Table 1. No statistical differences were found between the two groups. The population was composed of old adults (mean age was 67 years vs 68 years for DnE and DE, respectively), about ¾ males, overweight/obese subjects (30 ± 6 kg/m^2^ vs 29 ± 4 kg/m^2^ for DnE and DE group, respectively) with a sub-optimal blood pressure control. LDL-cholesterol was slightly above target, and renal function was preserved. In terms of glucose control, fasting glucose and glycated haemoglobin were mildly elevated (7.6 mmol/L and 52 mmol/mol vs 7.8 mmol/L and 51 mmol/mol, respectively) with a disease duration inferior to ten years. The two groups did not differ for administered drugs. 17% suffered from a previous CV event.

**Table 1:** Baseline clinical and biochemical characterization of the study population

	DnE	DE	*p*
Age (years)	67 ± 6	68 ± 7	ns
Male (%)	75	71	ns
BMI (Kg/m^2^)	30 ± 6	29 ± 4	ns
Alcohol intake low/medium/high (%)	50/38/12	54/38/8	ns
T2D duration (years)	7 [4; 10.5]	7.5 [4; 12]	ns
Oral antidiabetic drugs (%)	96	100	ns
Insulin (%)	17	25	ns
Systolic blood pressure (mmHg)	140 [131; 153]	145 [136; 159]	ns
Diastolic blood pressure (mmHg)	83 [73; 90]	82 [73; 90]	ns
Fasting glucose (mmol/L)	7.6 [6.0; 9.1]	7.8 [6.1; 10.7]	ns
HbA1_c_ (mmol/mol)	52 [45; 56]	51 [44; 61]	ns
eGFR (ml/min/1·73m^2^)	85 [77; 92]	87 [73; 98]	ns
CV events (%)	17	17	ns
Total cholesterol (mmol/L)	4.3 [3.9; 5.2]	4.3 [3.7; 5.1]	ns
HDL cholesterol (mmol/L)	1.1 [1.0; 1.4]	1.1 [1.0; 1.3]	ns
LDL cholesterol (mmol/L)	2.4 [1.8; 2.6]	2.5 [1.7; 3.3]	ns
Triglycerides (mmol/L)	1.7 [1.3; 2.3]	1.5 [1.1; 1.8]	ns
ΣDEHP (μg/g creatinine)	21.0 [13.3; 31.0]	22.6 [14.9; 40.0]	ns
MEHP (μg/g creatinine)	3.6 [2.2; 4.9]	3.5 [2.2; 5.2]	ns
MEOHP (μg/g creatinine)	4.8 [2.7; 6.8]	5.0 [3.5; 8.5]	ns
MEHHP (μg/g creatinine)	13.6 [6.8; 18.5]	13.8 [8.2; 24.6]	ns
RMR1	4.8 [2.5; 5.5]	4.4 [2.5; 7.0]	ns
RMR2	0.4 [0.3; 0.4]	0.4 [0.3; 0.5]	ns

RMR_1_ represents the rate of MEHP hydroxylation to MEHHP. RMR_2_ represents the rate of MEHHP oxidation to MEOHP

Phthalates urinary levels were also very similar between the groups. In detail, the DEHP metabolites (both taken individually and as a whole) were slightly higher in the DE group, but these differences did not reach statistical significance. Also, urinary levels of phthalates metabolites or RMRs do not correlate with any measured anthropometric and biochemical parameters, neither in the general population nor in the two subgroups.

### Follow-up

**Fig. 2:**
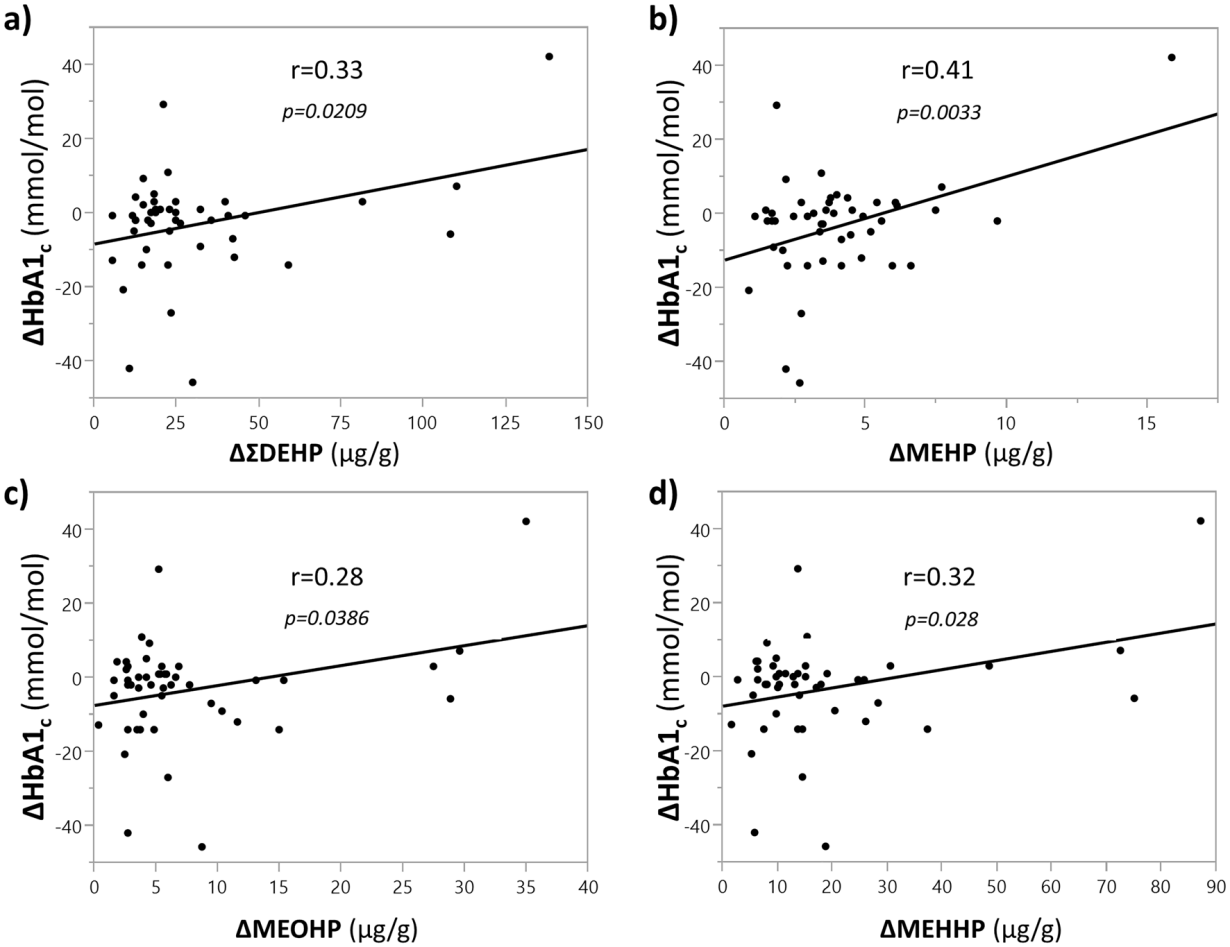
Correlation between baseline phthalates exposure and changes in glycated haemoglobin over the follow-up period

The mean follow-up duration was 19 ± 2 months. As expected by optimized standard routinary care, glucose control was stable/slightly improved over the follow-up. No changes in BMI, systolic and diastolic blood pressure and lipid serum profile were detected. This was also confirmed after stratification for edentulism status (Table 2). No sex-specific difference was observed. To explore the potential of phthalate’s exposure as a biomarker of metabolic derangement, we assessed the correlation between baseline phthalates’ exposure and all the observed parameters. Baseline phthalates exposure did not correlate with changes in BMI, systolic or diastolic blood pressure, fasting plasma glucose, eGFR, total cholesterol, LDL cholesterol, HDL cholesterol and triglycerides, RMR_1_ and RMR_2_. However, a positive and consistent correlation was observed between baseline levels of all the phthalates metabolites and HbA1_c_ variations (Fig. 2). In particular, the MEHP one was stronger (r = 0.41, p = 0.0033) followed by ΣDEHP (r = 0.33, p = 0.0209) and MEHHP (r = 0.32, p = 0.028), while MEOHP showed a weaker association (r = 0.28, p = 0.0386). Notably, all these correlations were confirmed even after adjusting for age, sex, BMI, edentulism status and administered drugs.

**Table 2:** Follow-up changes in the clinical and biochemical characterization of the study population

	DnE	DE	*p*
ΔBMI (Kg/m^2^)	− 0.6 [− 5.8; 2.8]	− 0.2 − .3; 4.1]	ns
ΔSystolic Blood Pressure (mmHg)	− 6 [− 14; 5]	− 8 [− 20; 15]	ns
ΔDiastolic Blood Pressure (mmHg)	− 2 [− 15; 11]	− 3 [− 16; 5]	ns
ΔFasting glucose (mmol/L)	− 0.1 [− 2.2; 0.9]	− 0.7 [− 3.8; 0.6]	ns
ΔHbA1_c_ (mmol/mol)	− 1 [− 8.25; 2.75]	− 2 [− 14; 3]	ns
ΔeGFR (ml/min/1·73m^2^)	0.8 [− 3.0; 4.2]	1.5 [− 2.4; 5.0]	ns
ΔTotal cholesterol (mmol/L)	0 [− 1.1; 1]	− 0.5 [− 1.4; 0.9]	ns
ΔHDL cholesterol (mmol/L)	0 [− 0.2; 0.3]	0.2 [− 0.1; 0.4]	ns
ΔLDL cholesterol (mmol/L)	− 0.1 [− 0.5; 0.9]	− 0.8 [− 1.4; 0.8]	ns
ΔTriglycerides (mmol/L)	− 0.2 [− 0.8; 0.3]	− 0.2 [− 0.6; 0.8]	ns

## Discussion

### Main findings

In our small single-centre pilot study performed in subjects with type 2 diabetes, we explored for the first time the relationship between phthalates and edentulism, showing that the two parameters were not associated. We also investigated, for the first time in adult humans in a longitudinal setting, the harmful potential of phthalates in terms of biomarkers of cardiometabolic derangement. We report that higher phthalates’ exposure was associated with HbA1_c_ worsening over time, and this association was confirmed for DEHP and its metabolites, both taken individually and as a whole.

### Phthalates and edentulism

We explored for the first time the relationship between phthalates and edentulism in humans, reporting the lack of an association. Dentistry diseases are recently raising interest from cardiologists and metabolic physicians due to the now recognized evidence of a relationship with cardiometabolic risk [29, 30]. Phthalates are also affirming as novel potential biomarkers for cardiometabolic health [10, 31]. Indeed, minimal quantities are sufficient to disrupt metabolic and endocrine pathways [32, 33]; therefore, it is essential to investigate an eventual involvement in oral medicine disease and a potential synergic detrimental effect on CV risk and metabolic derangement. However, to date, only one large epidemiologic study verified an association between mono-n-methyl phthalate and periodontal disease, but only in current smokers [34]. The relevance of investigating this association also lies in the fact that some actual and proposed devices for treating periodontal disease contain phthalates [35, 36]. In the present study, higher exposure to phthalates was not associated with edentulism. It must be pointed out that this is a cross-sectional observation limited to a population of type 2 diabetes individuals.

### Phthalates and glucose control

In our study, we report for the first time the association of baseline phthalate exposure with an increase in HbA1c over a mid-term follow-up in adult subjects with type 2 diabetes. It should be reported that only a few longitudinal studies are available in the current literature, displaying unclear or conflicting results. However, they focus on infancy [37], childhood [38], or are limited to the pregnancy period [39]. These results’ novelty also leads to its implicit therapeutic and prognostic implications. HbA1c was associated with phthalates in a cross-sectional setting in Chinese subjects with and without diabetes [40]. The correlations reported by Duan et al. were, however, relatively weak. MEHP was associated with fasting glucose only in the male sex. A positive association was reported only between MEHHP and HbA1c levels, with a peculiar relationship of specific phthalates with HbA1c in subgroups (MEOHP and ΣDEHP in BMI < 25 kg/m^2^ subgroup). In our study, we did not detect an association of phthalates with changes in fasting glucose, albeit, as expected, we did detect an association between changes in fasting glucose and changes in glycated haemoglobin (r = 0.5, p = 0.002). Also, even if the adjustment for confounders did not change the significance of our results, we cannot exclude that we are missing to detect putative sex-, age- or BMI-specific associations due to the small size of our cohort. However, it is interesting to observe that in our study group, in the cross-sectional observation, phthalates exposure was not related to fasting glucose or HbA1c. At the same time, the association with the glycated haemoglobin variation was more robust than the one described by Duan and consistent for all the phthalates. Although phthalates are non-persistent pollutants, they are indeed recognized as endocrine disruptors, possibly associated with the development of cardiometabolic disease, as we also recently reported [10, 26]. We thus infer that phthalates might be biomarkers with a more robust predictive, rather than descriptive, power. It should also be discussed that MEHP shows the strongest association, consistent with other reports [10]. A pathophysiologic explanation might be found in the MEHP capacity of stimulating glucose-induced insulin secretion, thus overburdening the β-cell and reducing its viability [41]. We did not see any correlation with the RMRs. However, our population was adult, which might support a more significant contribution of RMRs in children, in which RMRs are higher and thus probably more sensible [28, 42]. Also, phthalates are associated with low-grade inflammation [43], an established driver of metabolic derangement [11]. The implication of these findings lies in the capacity of the phthalates to hinder therapy effects and sustain the residual CV risk, a significant issue of the healthcare system nowadays [44]. Being phthalates related to poor socio-economic conditions [4–6], these associations might also partially justify the poorer glucose control found in this population, as reported by a recent large meta-analysis [45].

### Limitations

Our study comes with several limitations. First, the small sample size might have hindered the effect’s appreciation. However, it should be commented that, as previously stated, our findings are solid and consistent across all the phthalates examined. Also, the duration of the follow-up might be regarded as relatively short. It should, however, be recognized that this is the first study to assess phthalate exposure as a predictor of deranged metabolic control in longitudinal research in adult humans with T2D. We have to acknowledge also that, though rather evident, the lack of association with edentulism is reported with the limits of a cross-sectional observation. No new incident edentulism was observed, but this is obviously due to the duration of the follow-up.

Moreover, we have to discuss that we did not detect sex-specific effects; however, when conducting separate analyses for men and women, our study was not enough powered, so no sex-specific consideration can be deducted from our work. We are also aware that some oral drugs may contain phthalates and that their level might vary a lot between different medications [20, 21]. We do not investigate any association with specific medicines due to their extreme variety compared to the small, simple size. We also have to report that, at the baseline visit, we did not inquire the participants for information on source-specific exposure to phthalates (as different nutritional habits involving an abundant use of packaged food). However, we strongly instructed them on the potentially harmful effect of phthalates and provided them with written memoranda showing them what to avoid to minimize their exposure to these environmental pollutants. It should be noted that the investigation of a specific exposure source was beyond the study's scope, as we aimed to explore how baseline levels of exposure might longitudinally affect the cardiometabolic phenotype. Finally, we lack information on albuminuria, a parameter that we know to be related to both CV risk and phthalates [10].

## Conclusions

We can conclude that, among a small cohort of patients with type 2 diabetes, phthalates exposure predicts the worsening of glucose control. Moreover, we do not report any association between phthalates’ exposure and edentulism. Our small observation feeds the research field of novel biomarkers of cardiometabolic derangement. However, our study must be minded as a pilot and hypothesis-generating study. Larger trials with a long time of observation and a more detailed biochemical characterization are needed to confirm the relevance of our findings and assess their potential aid in the precise individual risk stratification.

## Data Availability

All data are available to qualified investigators upon reasonable request by contacting the corresponding author.

## Abbreviations

BMI: Body mass index
CV: Cardiovascular
DEHP: 2-Ethylhexyl-phthalate
eGFR: Estimated glomerular filtration rate
MEHP: Mono 2-ethylhexyl phthalate
MEOHP: Mono-2-ethyl-5-oxohexyl phthalate
MEHHP: Mono 2-ethyl-5-hydroxyhexyl phthalate
RMR: Relative metabolic rate
T2D: Type 2 diabetes
U-HPLC/LCMS: U-High Performance Liquid Chromatography/Liquid Chromatography Mass Spectrometry

## References

[1] Mariana M, Feiteiro J, Verde I, Cairrao E (2016). The effects of phthalates in the cardiovascular and reproductive systems: a review. Environ Int.

[2] Wittassek M, Koch HM, Angerer J, Brüning T (2011). Assessing exposure to phthalates—the human biomonitoring approach. Mol Nutr Food Res.

[3] Cullen E, Evans D, Griffin C, Burke P, Mannion R, Burns D (2017). Urinary phthalate concentrations in mothers and their children in Ireland: Results of the DEMOCOPHES human biomonitoring study. Int J Environ Res Public Health.

[4] Park C, Hwang M, Baek Y, Jung S, Lee Y, Paek D (2019). Urinary phthalate metabolite and bisphenol A levels in the Korean adult population in association with sociodemographic and behavioral characteristics: Korean National Environmental Health Survey (KoNEHS) 2012–2014. Int J Hyg Environ Health.

[5] Casteleyn L, Dumez B, Becker K, Kolossa-Gehring M, Den Hond E, Schoeters G (2015). A pilot study on the feasibility of European harmonized human biomonitoring: Strategies towards a common approach, challenges and opportunities. Environ Res.

[6] Li N, Friedrich R, Maesano CN, Medda E, Brescianini S, Stazi MA (2019). Lifelong exposure to multiple stressors through different environmental pathways for European populations. Environ Res.

[7] Tait S, Carli F, Busani L, Buzzigoli E, Della Latta V, Deodati A (2020). Biomonitoring of Bis(2-ethylhexyl)phthalate (DEHP) in Italian children and adolescents: data from LIFE PERSUADED project. Environ Res.

[8] Runkel AA, Snoj-Tratnik J, Mazej D, Horvat M (2020). Urinary phthalate concentrations in the slovenian population: an attempt to exposure assessment of family units. Environ Res.

[9] Lind PM, Zethelius B, Lind L (2012). Circulating levels of phthalate metabolites are associated with prevalent diabetes in the elderly. Diabetes Care.

[10] Mengozzi A, Carli F, Biancalana E, Della Latta V, Seghieri M, Gastaldelli A (2019). Phthalates exposure as determinant of albuminuria in subjects with type 2 diabetes: a cross-sectional study. J Clin Endocrinol Metab.

[11] Mengozzi A, Pugliese NR, Chiriaco M, Masi S, Virdis A, Taddei S (2021). Microvascular ageing links metabolic disease to age-related disorders: the role of oxidative stress and inflammation in promoting microvascular dysfunction. J Cardiovasc Pharmacol.

[12] Romandini M, Baima G, Antonoglou G, Bueno J, Figuero E, Sanz M (2021). Periodontitis, edentulism, and risk of mortality: a systematic review with meta-analyses. J Dent Res.

[13] Taboza ZA, Costa KL, Silveira VR, Furlaneto FA, Montenegro R, Russell S (2018). Periodontitis, edentulism and glycemic control in patients with type 2 diabetes: a cross-sectional study. BMJ Open Diabetes Res Care.

[14] Parolini F, Biancalana E, Rossi C, Raggi F, Mengozzi A, Solini A (2021). Clinical and epigenetic determinants of edentulism in type 2 diabetic subjects referring to a tertiary center. J Diabetes Complications.

[15] Ruospo M, Palmer SC, Craig JC, Gentile G, Johnson DW, Ford PJ (2014). Prevalence and severity of oral disease in adults with chronic kidney disease: a systematic review of observational studies. Nephrol Dial Transplant.

[16] Yu NH, Shin AR, Ahn SV, Song KB, Choi YH (2021). Estimation and change of edentulism among the Korean population: Korea National Health and Nutrition Examination Survey 2007–2018. Epidemiol Health.

[17] Raskiliene A, Kriaucioniene V, Siudikiene J, Petkeviciene J (2020). Self-reported oral health, oral hygiene and associated factors in lithuanian adult population, 1994–2014. Int J Environ Res Public Health.

[18] Delgado-Angulo EK, Mangal M, Bernabé E (2019). Socioeconomic inequalities in adult oral health across different ethnic groups in England. Health Qual Life Outcomes.

[19] Shiue I (2015). Urinary heavy metals, phthalates, phenols, thiocyanate, parabens, pesticides, polyaromatic hydrocarbons but not arsenic or polyfluorinated compounds are associated with adult oral health: USA NHANES, 2011–2012. Environ Sci Pollut Res Int.

[20] Chung BY, Choi SM, Roh TH, Lim DS, Ahn MY, Kim YJ (2019). Risk assessment of phthalates in pharmaceuticals. J Toxicol Environ Health A.

[21] Ennis ZN, Broe A, Pottegård A, Ahern TP, Hallas J, Damkier P (2018). Cumulative exposure to phthalates from phthalate-containing drug products: a Danish population-wide study. Br J Clin Pharmacol.

[22] Husøy T, Andreassen M, Hjertholm H, Carlsen MH, Norberg N, Sprong C (2019). The Norwegian biomonitoring study from the EU project EuroMix: Levels of phenols and phthalates in 24-hour urine samples and exposure sources from food and personal care products. Environ Int.

[23] Sundararaj SC, Thomas MV, Peyyala R, Dziubla TD, Puleo DA (2013). Design of a multiple drug delivery system directed at periodontitis. Biomaterials.

[24] McGarry TJ, Nimmo A, Skiba JF, Ahlstrom RH, Smith CR, Koumjian JH (1999). Classification system for complete edentulism. The American College of Prosthodontics. J Prosthodont.

[25] ADA (2020). 2 Classification and diagnosis of diabetes: standards of medical care in diabetes—2020. Diabetes Care.

[26] Mengozzi A, Carli F, Guiducci L, Parolini F, Biancalana E, Gastaldelli A (2021). SGLT2 inhibitors and thiazide enhance excretion of DEHP toxic metabolites in subjects with type 2 diabetes: a randomized clinical trial. Environ Res.

[27] Koch HM, Bolt HM, Preuss R, Angerer J (2005). New metabolites of di(2-ethylhexyl)phthalate (DEHP) in human urine and serum after single oral doses of deuterium-labelled DEHP. Arch Toxicol.

[28] Song NR, On JW, Lee J, Park JD, Kwon HJ, Yoon HJ (2013). Biomonitoring of urinary di(2-ethylhexyl) phthalate metabolites of mother and child pairs in South Korea. Environ Int.

[29] Liccardo D, Cannavo A, Spagnuolo G, Ferrara N, Cittadini A, Rengo C (2019). Periodontal disease: a risk factor for diabetes and cardiovascular disease. Int J Mol Sci..

[30] Carrizales-Sepúlveda EF, Ordaz-Farías A, Vera-Pineda R, Flores-Ramírez R (2018). Periodontal disease, systemic inflammation and the risk of cardiovascular disease. Heart Lung Circ.

[31] Mariana M, Cairrao E (2020). Phthalates implications in the cardiovascular system. J Cardiovasc Dev Dis..

[32] Tassinari R, Tait S, Busani L, Martinelli A, Valeri M, Gastaldelli A (2021). Toxicological assessment of oral Co-Exposure to Bisphenol A (BPA) and Bis(2-ethylhexyl) Phthalate (DEHP) in juvenile rats at environmentally relevant dose levels: evaluation of the synergic, additive or antagonistic effects. Int J Environ Res Public Health.

[33] Tassinari R, Tait S, Busani L, Martinelli A, Narciso L, Valeri M (2021). Metabolic, reproductive and thyroid effects of bis(2-ethylhexyl) phthalate (DEHP) orally administered to male and female juvenile rats at dose levels derived from children biomonitoring study. Toxicology.

[34] Emecen-Huja P, Li HF, Ebersole JL, Lambert J, Bush H (2019). Epidemiologic evaluation of Nhanes for environmental Factors and periodontal disease. Sci Rep..

[35] Vidal-Romero G, Zambrano-Zaragoza ML, Martínez-Acevedo L, Leyva-Gómez G, Mendoza-Elvira SE, Quintanar-Guerrero D (2019). Design and evaluation of pH-dependent nanosystems based on cellulose acetate phthalate, nanoparticles loaded with chlorhexidine for periodontal treatment. Pharmaceutics.

[36] Morkhade DM, Nande VS, Barabde UV, Patil AT, Joshi SB (2018). Design and evaluation of dental films of PEGylated rosin derivatives containing sparfloxacin for periodontitis. Drug Dev Ind Pharm.

[37] Navaranjan G, Takaro TK, Wheeler AJ, Diamond ML, Shu H, Azad MB (2020). Early life exposure to phthalates in the Canadian Healthy Infant Longitudinal Development (CHILD) study: a multi-city birth cohort. J Expo Sci Environ Epidemiol.

[38] Watkins DJ, Peterson KE, Ferguson KK, Mercado-García A, Tamayo y Ortiz M, Cantoral A (2016). Relating phthalate and BPA exposure to metabolism in peripubescence: the role of exposure timing, sex, and puberty. J Clin Endocrinol Metab.

[39] Gao H, Zhu BB, Huang K, Zhu YD, Yan SQ, Wu XY (2021). Effects of single and combined gestational phthalate exposure on blood pressure, blood glucose and gestational weight gain: a longitudinal analysis. Environ Int.

[40] Duan Y, Sun H, Han L, Chen L (2019). Association between phthalate exposure and glycosylated hemoglobin, fasting glucose, and type 2 diabetes mellitus: a case-control study in China. Sci Total Environ.

[41] Weldingh NM, Jørgensen-Kaur L, Becher R, Holme JA, Bodin J, Nygaard UC (2017). Bisphenol A is more potent than phthalate metabolites in reducing pancreatic β-cell function. Biomed Res Int.

[42] On J, Kim SH, Lee J, Park MJ, Lee SW, Pyo H (2021). Urinary di(2-ethylhexyl)phthalate metabolite ratios in obese children of South Korea. Environ Sci Pollut Res Int.

[43] Campioli E, Martinez-Arguelles DB, Papadopoulos V (2014). In utero exposure to the endocrine disruptor di-(2-ethylhexyl) phthalate promotes local adipose and systemic inflammation in adult male offspring. Nutr Diabetes.

[44] Paneni F, Diaz Cañestro C, Libby P, Lüscher TF, Camici GG (2017). The aging cardiovascular system: understanding it at the cellular and clinical levels. J Am Coll Cardiol.

[45] Bijlsma-Rutte A, Rutters F, Elders PJM, Bot SDM, Nijpels G (2018). Socio-economic status and HbA 1c in type 2 diabetes: a systematic review and meta-analysis. Diabetes Metab Res Rev.

